# Women’s experiences of pharmacological and non-pharmacological pain relief methods for labour and childbirth: a qualitative systematic review

**DOI:** 10.1186/s12978-019-0735-4

**Published:** 2019-05-30

**Authors:** Gill Thomson, Claire Feeley, Victoria Hall Moran, Soo Downe, Olufemi T. Oladapo

**Affiliations:** 1 School of Community Health & Midwifery, University of Central Lancashire, Preston, Lancashire PR1 2HE UK; 2 Development and Research Training in Human Reproduction (HRP), Department of Reproductive Health and Research, World Health Organization, 20 Avenue Appia, 1211 Geneva, Switzerland

**Keywords:** CERQual, Childbirth, Epidural, Labour, Massage, Opiates, Opioids, Pain relief, Qualitative, Relaxation

## Abstract

**Background:**

Many women use pharmacological or non-pharmacological pain relief during childbirth. Evidence from Cochrane reviews shows that effective pain relief is not always associated with high maternal satisfaction scores. However, understanding women’s views is important for good quality maternity care provision. We undertook a qualitative evidence synthesis of women’s views and experiences of pharmacological (epidural, opioid analgesia) and non-pharmacological (relaxation, massage techniques) pain relief options, to understand what affects women’s decisions and choices and to inform guidelines, policy, and practice.

**Methods:**

We searched seven electronic databases (MEDLINE, CINAHL, PsycINFO, AMED, EMBASE, Global Index Medicus, AJOL), tracked citations and checked references. We used thematic and meta-ethnographic techniques for analysis purposes, and GRADE-CERQual tool to assess confidence in review findings. We developed review findings for each method. We then re-analysed the review findings thematically to highlight similarities and differences in women’s accounts of different pain relief methods*.*

**Results:**

From 11,782 hits, we screened full 58 papers. Twenty-four studies provided findings for the synthesis: epidural (*n* = 12), opioids (*n* = 3), relaxation (*n* = 8) and massage (*n* = 4) – all conducted in upper-middle and high-income countries (HMICs). Re-analysis of the review findings produced five key themes. ‘*Desires for pain relief’* illuminates different reasons for using pharmacological or non-pharmacological pain relief. *‘Impact on pain’* describes varying levels of effectiveness of the methods used. ‘*Influence and experience of support’* highlights women’s positive or negative experiences of support from professionals and/or birth companions. ‘*Influence on focus and capabilities’* illustrates that all pain relief methods can facilitate maternal control, but some found non-pharmacological techniques less effective than anticipated, and others reported complications associated with medication use. Finally, ‘*impact on wellbeing and health’* reports that whilst some women were satisfied with their pain relief method, medication was associated with negative self-reprisals, whereas women taught relaxation techniques often continued to use these methods with beneficial outcomes.

**Conclusion:**

Women report mixed experiences of different pain relief methods. Pharmacological methods can reduce pain but have negative side-effects. Non-pharmacological methods may not reduce labour pain but can facilitate bonding with professionals and birth supporters. Women need information on risks and benefits of all available pain relief methods.

## Plain english summary

For most women, labour pain is the most severe pain they will ever experience. Women regularly use medications and/or natural methods for labour pain relief. We searched for published studies on women’s views and experiences of epidurals, opioid injections such as pethidine, relaxation and massage techniques. We included 24 good quality studies, all from high and middle-income countries (HMICs). We developed review findings for each method. We then examined differences and similarities in women’s experiences of different pain relief methods. *‘Desires for pain relief’* highlights the different reasons women give for choosing medications or other approaches. *‘Impact on pain’* describes how the techniques either were or were not effective in reducing labour pain. ‘*Influence and experience of support’* highlights women’s experiences of positive or negative support from professionals and/or birth companions. ‘*Influence on focus and capabilities’* describes that while all pain relief methods could help women feel in control, some found the natural methods to be less effective than anticipated, and others disliked complications they experienced after using medication. ‘*Impact on wellbeing and health’* reports that whilst women could be satisfied with their pain relief method, some who used medication felt guilty, whereas women taught relaxation techniques often continued to use these methods with benefits for women/infants. These findings highlight that women have mixed experiences of different pain relief methods, and that they need information on risks and benefits of all available pain relief methods.

## Background

For many women, the pain they experience during labour and childbirth will be the most severe form of pain they have ever experienced [1]. Pain is considered to be a unique and individual experience. Accounts vary from pleasurable to unbearable, with both extremes sometimes reported to occur concurrently [2, 3]. Women’s perceptions of pain are affected by physiological (e.g. birth position) and/or psychological issues (e.g. fear, anxiety) [4], and the quality of the woman-provider relationship [2]. Some women cope well with labour pain without any intervention, whereas others require pharmacological and/or non-pharmacological methods for pain relief [5]. Effective pain management has become an essential component of the care plan for childbearing women.

Globally, pharmacological interventions are frequently used during labour and childbirth. Epidural analgesia is regarded to be an effective form of pain relief [5], however, it is not necessarily associated with a positive experience of birth [6]. In addition, this form of pain relief is expensive, can decrease women’s feelings of control, delay second stage of labour and increase the likelihood of further interventions (such as instrumental birth, and caesarean section) [6]. Another commonly applied pharmacological method is opioids, particularly pethidine [7]. This method can help women relax and cope with pain due to strong uterine contractions, but unlike an epidural, enable women to retain mobility. However, unwanted side effects include nausea, sedation and a negative impact on women’s ability to safely breastfeed their infant [8], and multiple doses can lead to the accumulation of metabolites, such as normeperidine, associated with narcotic-induced depression in infants [9].

Non-pharmacological pain relief methods associated with relaxation and massage are referred to as mind-body interventions [5]. Relaxation methods such as yoga, music and breathing techniques, and different forms of massage (e.g. shiatsu, reflexology) are designed to induce calm and to distract/alleviate pain in labouring women. Trials of relaxation techniques during labour have reported less intense pain, increased satisfaction with pain relief and childbirth [5], and lower rates of assisted vaginal birth [10], without any adverse outcomes [5]. However, there was a large variation in how these relaxation techniques were applied [11]. A Cochrane systematic review [5] identified relaxation and massage methods as safe and non-invasive, based on low quality evidence.

In 2016 the World Health Organisation recognised the importance of shaping new antenatal guidelines through finding out what mattered to pregnant women [12]. This work involved quantitative systematic reviews to inform the safety, efficacy and cost of antenatal interventions, together with qualitative evidence syntheses relating to the views and experiences of service users and service providers to inform the values, equity, and acceptability components of each guideline recommendation. A similar mixed evidence approach has been used for the forthcoming WHO intrapartum guidelines for healthy women and infants.

This study was conducted to support guidance on pain relief as part of evidence base preparation for the WHO recommendations on intrapartum care for a positive childbirth experience. A qualitative evidence synthesis that comprised four separate searches into women’s views and experiences of pharmacological (epidural and opioids) and non-pharmacological (relaxation and massage techniques) pain relief methods used during labour and childbirth was undertaken. Here we report the review findings of each pain relief method. Further analysis of the review findings to highlight similarities and differences in women’s experiences of pharmacological or non-pharmacological pain relief methods is also presented.

## Methods

This review was informed by four separate searches into women’s experiences of using epidurals, opioids, massage and relaxation. Our methods incorporated a pre-designed search strategy, quality appraisal techniques [13], and an assessment of confidence in the findings using the GRADE-CERQual tool [14]. Data analysis was carried out using thematic [15] and meta-ethnographic techniques [16].

### Reflexivity

Quality standards for qualitative research and qualitative evidence syntheses ideally include author reflexivity prior and during the research process [17]. Therefore, the authors considered their views and opinions on methods of pain relief from women’s perspectives. All authors believe in endeavours that support women to have a positive birth experience. While all authors have concerns about rising intervention rates, we consider it important for women to have their individual needs met, whether that is access to pharmacological pain relief, or not. We believe that women’s individualised needs are best served through respectful, meaningful relationships with caregivers who are able to respond and deliver pain relief methods as required.

### Search strategy

Searches were developed using a population, exposure and outcome (PEO) strategy (see table one). Search terms related to study type was added if the hits from the initial search exceeded 1000. These terms were designed to identify studies that had a specific qualitative focus or mixed-methods studies that included a substantial qualitative component. We developed search terms following a priori scoping exercises in titles and abstracts, adapted for specific database architecture. Table 1 details all the search terms. We searched MEDLINE, CINAHL, PsycINFO, AMED, EMBASE, Global Index Medicus, and AJOL (for studies conducted in Africa). Additional search strategies included citation tracking and reference checking.

**Table 1:** Details of PEO search terms used in all four searches

Population	Woman or women* or mother* or mum* or maternal
Exposure – intrapartum	Intrapartum or intra-partum or intra-natal or intranatal or birth* or childbirth or labour* or labor* or parturition
Exposure – epidural	Epidural* or epidural analgesia or epidural anesthesia or epidural anaesthesia or spinal-epidural or spinal epidural or spinal anaesthesia or spinal anesthesia or spinal analgesia or analgesia or anaesthsia or anesthesia
Exposure - opioids	Opioid* or pethidine or meperidine or Demerol or diamorphine or nalbuphine or butorphanol or meptazinol or pentazocine or fentanyl or remifentanil or tramadol or opiates
Exposure – massage	Massage or reflexology or zero balancing or trigger point or therapeutic touch or shiatsu or osteopath or neuromuscular massage or neuromuscular facilitation or myotherapy or myofacial release or musculo*skeletal therapy or manual therapy or deep tissue massage or cranio*sacral therapy or chiropractic* or bio*energy therapy or acupressure or tui na or compress*
Exposure - relaxation	Yoga or meditation or imagery or visuali*ation or breathing exercise* or music or audio*analgesia or progressive muscle relaxation or breathing technique* or psycho*prophylaxis or guided imagery or mindfulness
Outcomes	View* or experienc* or perspective* or perception* or opinion* or belief* or assum* or understand* or encounter* or attitude* or prefer* or feel* feasibil* or acceptab* or help* or meaning* or value*
Study type (only used if the hits using the terms above exceeded 1000)	Qualitative or interview* or “focus group*” or ethnograph* or phenomenolog* or “grounded theory”

### Inclusion/exclusion criteria

No language restriction was imposed. Papers not in English were translated via Google translate where possible. Only papers that reported the views and experiences of healthy women who had experience of using at least one of the four methods of interest were included. Studies published before 1996 were excluded, to ensure the findings reflect current service provision, since epidural analgesia, in particular, was not commonly used before the mid 1990s. We also excluded studies of hypothetical views of pain relief, the views and experiences of partners or healthcare providers, and populations of women with complications.

### Study selection

Searches were carried out during June–July 2017. GT screened the initial hits (title and abstracts) against the inclusion criteria. Full texts were blind screened by GT and CF, and inclusion agreed by consensus. Where there was disagreement, a third author (VHM) screened the text, after which final consensus was reached.

### Quality assessment

All eligible papers were quality appraised using an instrument developed by Walsh and Downe [17] and modified by Downe, Walsh, Simpson & Steen [13]. The framework was used to assess studies against pre-defined criteria, and then allocate a score from A-D (Table 2). Only studies that scored C- or higher were to be included in the final analysis.

**Table 2:** Scoring criteria for quality appraisal

A: No, or few flaws. The study credibility, transferability, dependability and confirmability are high; B: Some flaws, unlikely to affect the credibility, transferability, dependability and/or confirmability of the study; C: Some flaws that may affect the credibility, transferability, dependability and/or confirmability of the study. D: Significant flaws that are very likely to affect the credibility, transferability, dependability and/or confirmability of the study.	

### Data analysis

Data analysis was carried out in two stages.

#### Stage one

We used a general thematic approach [15], meta-ethnography [16], and CERQual (Confidence in the Evidence from Reviews of Qualitative Research [14]. All analytical stages were undertaken individually and then together by GT and CF. All decisions were shared and discussed with all members of the review team. Data were logged on excel.

We undertook an initial reading of each paper to identify relevant sections of text reported in quote material, author themes and statements (‘first order constructs’) [15]. These were then grouped into descriptive themes (‘second order constructs’). During this process, we used meta-ethnographic [15] techniques to identify what was similar (‘reciprocal data’) across the included studies, and what contradicted our emerging analysis (‘refutational data’). We reframed the themes as the analysis progressed, to account for both reciprocal and refutational data as we identified it. We then translated the themes into Summary of Findings statements. These were assessed for confidence using the GRADE-CERQual [14] approach, in which the studies that contribute to each summary of findings statement are assessed for methodological limitations, relevance to the review question, coherence in terms of whether clear patterns across the studies could be identified and adequacy with regard to the coverage of the elements of the review finding, and of the geographical area/contexts it related to [14]. Following an assessment of the four criteria, each review finding was graded for confidence on a scale of ‘high’, ‘moderate’, ‘low’, or ‘very low’.

#### Stage two

The results of the first stage indicated that there were key areas of convergence and divergence in women’s experiences of different pain relief methods. We considered that a comparative focus would enable a deeper understanding of how varying methods of pain relief are experienced, and how those experiences are internalised psychologically over time. During this phase, we used a basic thematic approach [14] to re-analyse all the first-order constructs into key themes that compared and contrasted women’s accounts of different pain relief methods. Analysis was initially undertaken by GT and CF, with all decisions shared and agreed by all authors.

## Results

**Fig. 1:**
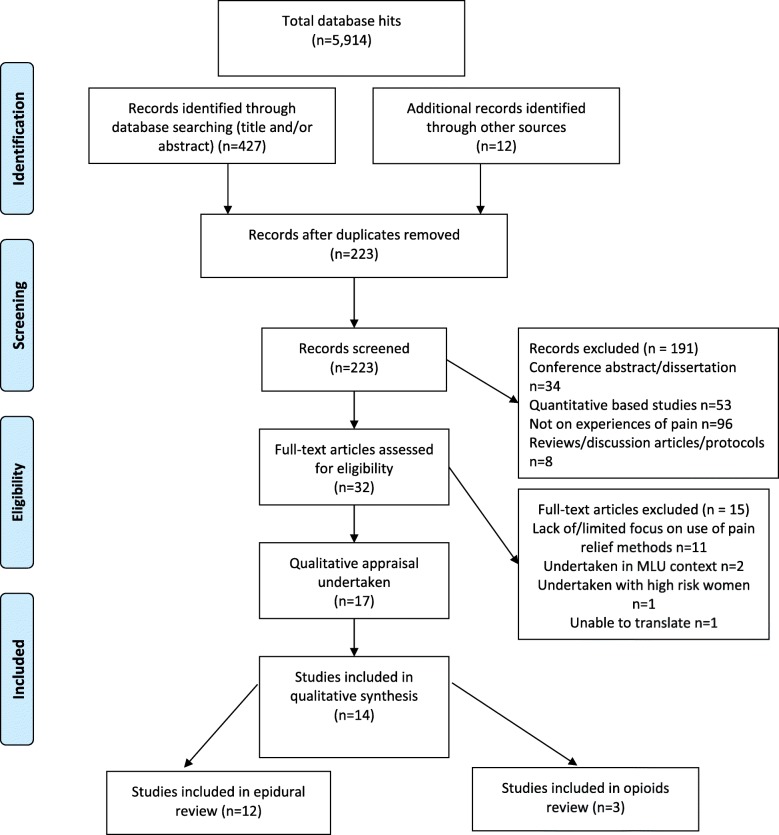
PRISMA Diagram Epidurals and Opioids

The pharmacological pain relief searches generated 5914 hits (see Fig. 1 for PRISMA). Following screening and quality appraisal 12 studies met inclusion criteria for the epidural review (*n* = 1507 women). Studies were undertaken in USA (*n* = 7), UK (*n* = 2), Denmark (*n* = 1), New Zealand (*n* = 1) and Canada (n = 1)). Three studies were included in the opioids review (*n* = 412 women) undertaken in the UK (n = 1), Australia (n = 1) and South Africa (n = 1). One of the studies contained data relevant to both reviews [18]. One study was unable to be translated [19]. We also excluded three studies that scored a D rating at quality appraisal [20–22]. The included studies were of mixed-methods (*n* = 1), general qualitative (*n* = 7) or phenomenological (*n* = 1) designs, and five collected qualitative data through open text questions on a survey.

**Fig. 2:**
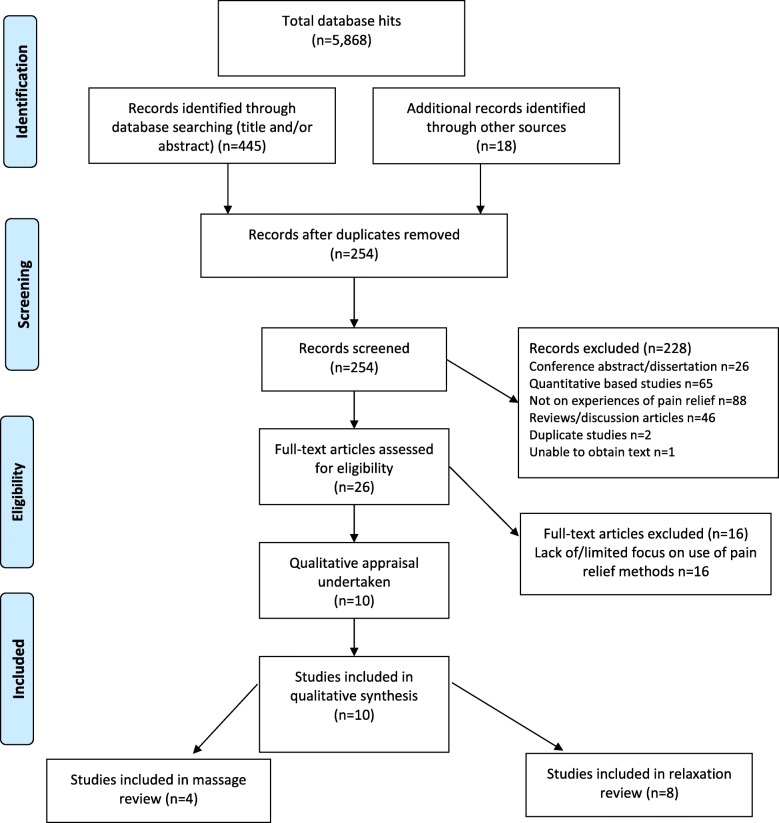
PRISMA Diagram Massage and Relaxation

The non-pharmacological review generated 5868 hits (see Fig. 2 for PRISMA). Following screening and quality appraisal, four studies were included in the massage review (*n* = 94 women). Studies were undertaken in Australia (*n* = 1), Brazil (n = 1), UK (n = 1) and Sweden (n = 1). Eight studies were included in the relaxation review (*n* = 99 women) undertaken in Australia (*n* = 2), Brazil (n = 2), Turkey (*n* = 1), Canada (n = 1) and USA (n = 2). Two contained data that were relevant to both reviews [23, 24]. Two of the studies translated using Google translate were comprehensible in English, and suitable for inclusion [25, 26]. The included studies were of mixed-methods (*n* = 3), general qualitative (n = 3) or phenomenological (*n* = 1) designs and the remaining studies collected qualitative data as part of a feasibility study (n = 1) or through open text survey questions (*n* = 2).

Study characteristics and quality ratings for the four pain relief methods are presented in Table 3. Apart from three studies undertaken in an upper-middle income country (Brazil (n = 2), South Africa (n = 1)) the remaining studies took place in a high-income context.

**Table 3:** Study characteristics and quality ratings for all included studies

Study Code & Authors	Date	Country	Resource	No of Participants	Parity	Type of pain relief	Primary aim/focus	Design	Qualitative data collection method	Quality Rating
Yoshioka, Yeo & Fetters, 2012 [29]	2012	USA	High	17 women	Primips & multips	Epidural	To understand importance of cultural barriers in use of epidurals for Japanese women living in USA	Mixed methods (survey/interview)	Interviews	C
Morris & Schulman, 2014 [35]	2014	USA	High	83 women	Primips & multips	Epidural	To explore organisational processes that may lead to racial disparity in epidural use and regional anaesthesia failure in labour and birth	Qualitative (unspecified)	Interviews	C+
Larkin, Begley & Devane, 2017 [18]	2017	Republic of Ireland	High	531 completed DCE - 291 provided qualitative comments	Not reported	Epidural and Opioids	Women’s preferences during childbirth/labour	Discrete choice experiment	Open ended survey questions	C+
Jepsen & Keller, 2014 [32]	2014	Denmark	High	9 women and 8 midwives	Primips	Epidural	How women experience being in labour with epidural analgesia and what kind of midwifery care do they consequently need	Phenomenology	Field observations and interviews	B+
Jantjes, Strumpher & Kotze, 2007 [37]	2007	South Africa	Upper Middle	9 women	Primips	Opioids	To explore/describe the childbirth experience of first-time mothers who received opioids during the first stage of labour	Qualitative (uses Kotze’s Nursing Accompaniment Theory)	Interviews	C+
Doering, Patterson & Griffiths, 2014 [34]	2014	New Zealand	High	13 women	Primips & multips	Epidural	To explore how Japanese women in New Zealand respond to the use of pharmacological pain relief in labour	Qualitative (unspecified)	Interviews/focus group	B+
Hidaka & Callister, 2012 [36]	2012	USA	High	9 women	Primips	Epidural	To understand the birth experiences of women using epidural analgesia for pain management	Qualitative (unspecified)	Interviews	B+
Angle, Landy, Charles, Yee, Watson, Kung, Kronberg, Halpern, Lam, Ming & Streiner, 2010 [33]	2010	Canada	High	28 women	Primips & multips	Epidural	To explore women’s experiences and perspectives of neuraxial analgesia for tool development	Qualitative (unspecified)	Interviews/focus groups	B+
Lally, Thomson, MacPhail & Exley, 2014 [31]	2014	UK	High	23 women	Primips & multips	Epidural	To explore how women can be better supported in preparing for and making decisions during pregnancy and labour regarding pain management	Qualitative (unspecified)	Interviews	B-
Dillaway & Brubaker, 2006 [28]	2006	USA	High	60 women	Not reported	Epidural	To analyse findings from two separate qualitative studies using an intersectionality framework to compare experiences of two different samples of birthing women (White, middle to upper class and African American teenagers)	Qualitative using intersectionality framework	Interviews	B
Attanasio, Kozhimannil, Jou, McPherson & Camann, 2015 [40]	2015	USA	High	1573 women surveyed (914 women provided comments in relation to opioid use)	Primips & multips	Epidural	To explore patient experience of neuraxial analgesia among a national sample of US mothers	Survey	Open ended survey questions	B-
Fleet, Jones & Belan, 2017 [41]	2017	Australia	High	112 women	Primips & multips	Opioids	RCT to compare the efficacy of fentanyl administered via different routes, with thestandard practice of intramuscular (IM) pethidine use for women in labour.	RCT study	Open ended survey questions	B-
Goldberg & Shorten, 2014 [30]	2014	USA	High	14 women, 13 labour nurses and 7 obstetricians/gynaecologists	Primips	Epidural	To explore differences between patient and provider perceptions of decision-making regarding epidural analgesia use during labor and birth	Survey	Open ended survey questions	B-
Heinze & Sleigh, 2003 [27]	2003	USA	High	46 women	Primips & multips	Epidural	To explore differences between women choosing to labour without medication and those choosing to labour with an epidural	Survey	Open ended survey questions	C
Levett, Smith, Bensoussan & Dahlen, 2016 [24]	2016	Australia	High	13 women, 7 partners and 12 midwives	Primips	Complementary medicine antenatal education course (including relaxation techniques and massage)	To gain insights into the experiences of women, partners and midwives who participated in the intervention	RCT study (interviews with intervention arm only)	Interviews/focus groups	B+
Miquelutti, Cecatti & Makuch, 2013 [23]	2013	Brazil	Upper-Middle	21 women (11 in intervention arm)	Primips	Systematized antenatal programme (including relaxation and massage techniques)	To describe the experience of labour and delivery as reported by women who participated and women who did not participate in an antenatal program of preparation.	RCT study (interviews with women who did/did not access the intervention)	Interviews	B+
Browning, 2000 [42]	2000	Canada	High	11 women	Primips	Music therapy (relaxation)	To explore experiences of the music group intervention	Qualitative (unspecified)	Interviews	C-
Kimber, 1998 [45]	1998	UK	High	50 women and partners	Primips & multips	Massage	To undertake an evaluation of the therapist’s massage service	Evaluation survey	Open ended survey questions	C-
Andren & Lundgren, 2005 [25]	2005	Sweden	High	10 women	Primips	Massage	To describe women’s experiences of tactile massage during the latent phase of labour	Phenomenology -	Interviews	A-
Fisher, Hauck, Bayes & Byrne, 2012 [39]	2012	Australia	High	12 mothers & 7 birth partners	Primips	Mindfulness intervention (relaxation)	To explore participant experiences of the mindfulness intervention	Feasibility study	Focus groups	B+
Klimi et al., 2011 [26]	2011	Turkey	Upper Middle	3 women	Primips	Music therapy (relaxation)	To explore how the music and other sounds can affect the experience of childbirth.	Qualitative (unspecified)	Interviews	C+
Tabarro, de Campos, Galli, Novo & Pereira, 2010 [43]	2010	Brazil	Upper Middle	12 women	Primips & multips	Music therapy (relaxation)	To verify and describe the effects of individually selected songs during the labour.	Qualitative (unspecified)	Interviews	C+
Duncan & Bardacke, 2010, [38]	2010	USA	High	27 women	Primips & multips	Mindfulness intervention (relaxation)	to describe the changes in the dimensions of the stress and coping process observed in pregnant women participating in the intervention with their partners during their third trimester of pregnancy.	Mixed-method observational pilot study	Interviews	B+
Pierce, 2001 [44]	2001	USA	High	76% return rate - but no denominator provided	No details	Toning (relaxation)	To undertake an evaluation of the therapist’s music/toning service	Evaluation survey	Open ended survey questions	C-

The Summary of Findings and CERQual ratings for each pain relief method from stage one analysis are detailed in Table 4 (full CERQual assessments are available on request). The themes arising from stage two analysis are mapped to these Summary of Findings statements and are discussed in detail in the next section.

**Table 4:** Review findings and CERQual ratings

Review finding	Studies contributing to the review finding	CERQual Assessment	Theme
EPIDURALS			
1. Information and awareness influences women’s decisions to have an epidural: Women’s pre-birth decision for an epidural was influenced by a previous positive experience of epidural use, or through messages (positive or negative) from health professionals, members of their social networks or the media. In some occasions, the frequency of use of epidurals, and awareness of risks/perceived perceptions of safety were a positive influence on women’s decision (i.e. women from countries where epidural use is not the norm). Women felt reassured and relieved by knowing an epidural was available.	8 studies: [18, 28–30, 33–36]	Moderate	*Desires for pain relief*
2. Pre-existing desires for pain relief: Women expressed a desire for epidural due to reasons such as wanting a pain-free labour, a fear of pain and a desire to remain in control during labour	10 studies: [18, 27–35]	Moderate	*Desires for pain relief*
3. Pain relief as last resort: Women opted to have an epidural at a crucial point in their labour where the level of pain was unmanageable and/or feeling that the labour was out of their control.	7 studies: [27, 29, 32–36]	Low	*Desires for pain relief*
4. Fear of procedure and impact: Women expressed fears towards epidural use associated with pain at citing, potential ineffectiveness of the anaesthesia and negative implications for self and baby.	5 studies: [28, 29, 33, 35, 36]	Low	*Desires for pain relief*
5. Pressure and persuasion by professionals and others (for epidural use): Women were actively encouraged, persuaded or pressured to have an epidural by health professionals, messages received via antenatal classes and lack of options for non- pharmacological methods.	7 studies: [18, 27, 28, 31, 34, 35, 40]	Low	*Influence and experience of support*
6. Negative impact on physiological and psychological responses: Women experienced adverse responses associated with pain/complications associated with needle insertion, negative side effects, lack of mobility, feeling disconnected from the labour and birth, and negative impact on their capacity to give birth physiologically. Women experienced negative emotions associated with epidurals such as conflict, guilt, disappointment and a sense of failure.	6 studies: [27, 32–34, 36, 40]	Low	*Influence on focus and capabilities* *Impact on wellbeing and health*
7. Helped to facilitate positive labour and birth: Following an epidural women were able to relax, rest and to restore and renew their energy levels to enable them to cope and manage during labour. An epidural provided women with a sense of control where they could focus on labour signs and make decisions regarding progress. Patient controlled epidural was positively perceived and facilitated mobility. Women’s fears of epidural were not met and they were able to enjoy and actively participate in the birth with no/manageable side-effects. Some women felt that the epidural had enabled them to achieve a normal physiological birth	9 studies: [18, 27, 28, 30–33, 36, 40]	Moderate	*Influence on focus and capabilities* *Impact on wellbeing and health*
8. Positive impact on pain: An epidural provided effective and significant pain relief for some women. Women were able to feel connected to the birth without experiencing constant pain.	6 studies: [18, 27, 28, 30, 33, 36]	Low	*Impact on pain*
9. Supported in their choice: Women valued having an epidural as a choice for pain relief, being able to make their own decision about the use of an epidural, and to be supported in their choice (by health professionals and family members).	6 studies: [28–30, 33, 35, 40]	Low	*Influence and experience of support*
10. Lack of consent/information: Women were not always fully aware of the risks or benefits of epidural use.	5 studies: [28, 32, 34, 36, 40]	Low	*Influence and experience of support*
11. Ineffective pain relief: Some women continued to experience pain/breakthrough pain after epidural citing. In some occasions the epidural was provided too late, wore off too early or requests for ‘top-ups’ were denied.	4 studies: [27, 33, 35, 40]	Low	*Impact on pain*
OPIOIDS			
1. Pain relief as last resort: Women opted to have opioids at a crucial point in their labour where the level of pain was unmanageable.	1 study: [37]	Very low	*Desires for pain relief*
2. Positive impact on pain and labour: Opioids had a positive impact on pain, shortened and reduced the intensity of the contractions (pethidine and other forms of opioids) and with no side effects (pethidine and other forms of opioids). It increased the woman’s enjoyment and helped them to give birth (not referred to in relation of Pethidine).	1 study: [41]	Very low	*Impact on pain* *Impact on focus and capabilities*
3. Negative impact on physiological and psychological responses: Following Pethidine women experienced negative physiological (e.g. sickness, ‘groggy’, slow labour, disconnected from the labour, inability to push) and psychological (e.g. disappointment, inability to remember the birth) affects.	2 studies: [37, 41]	Very low	*Impact on focus and capabilities* *Impact on wellbeing and health*
4. Ineffective pain relief: Women continued to experienced pain due to the opioids being ineffective, provided too late or wore off too early. In some occasions, the pain was exacerbated (Pethidine only).	3 studies: [18, 37, 41]	Very low	*Impact on pain*
5. Lack of/insufficient support: Women were disappointed due to over-reliance on staff (due to need/desire for additional support or ongoing receipt of medication)	2 studies: [37, 41]	Very low	*Influence and experience of support*
6. Lack of information/consent: Women were not always fully aware of the route of administration or the risks of Pethidine use.	1 study: [37]	Very low	*Influence and experience of support*
MASSAGE			
1. Massage techniques facilitated labour coping skills: Women found that massage techniques were useful to enable them to cope and manage the labour process.	4 studies: [23–25, 45]	Low	*Impact on focus and capabilities*
2. Positive way to ‘work with the pain’: The use of massage techniques gave women an alternative method to deal with labour pain - women reported that massage techniques reframed their approach of managing pain through the positive concept of ‘working with the pain’.	3 studies: [24, 25, 45]	Low	*Impact on pain*
3. Positive impact upon sense of relaxation and control: Women reported that massage techniques enhanced relaxation and provided inner resources to remain calm and maintain self-control.	4 studies: [23–25, 45]	Low	*Impact on focus and capabilities*
4. Enhanced wellbeing: Women reported that massage techniques were beneficial to their wellbeing, including finding massage reassuring, positive, a means to overcome anxieties and provided a sense of safety during the birth.	3 studies: [23, 25, 45]	Low	*Impact on focus and capabilities* *Impact on wellbeing and health*
5. Enhanced participation of birth companions and health professionals: Women reported that taught massage techniques provided their birth companions with the tools to participate in labour preparation and during the birth. Additionally, when midwives performed the massage, this contributed to positive emotional and physical closeness.	3 studies: [24, 25, 45]	Low	*Influence and experience of support*
6. Ineffective pain relief: For a minority of women the techniques were not always effective in alleviating pain, or were negatively influenced by maternal position.	2 studies: [23, 45]	Very low	*Impact on pain* *Impact on focus and capabilities*
7. Valued variety of techniques: Where women were taught a range of techniques during the antenatal period, women valued the variety to adapt to their changing needs throughout labour.	1 study: [24]	Very low	*Impact on focus and capabilities*
RELAXATION			
1. Increased confidence approaching childbirth: Women valued being taught relaxation techniques during the antenatal period in readiness for labour. For some, this was considered effective in reconstructing fears of labour, for others it increased their feelings of confidence approaching childbirth	4 studies: [24, 26, 38, 42]	Low.	*Desires for pain relief*
2. Relaxation techniques facilitated labour coping skills: Women reported that the variety of techniques previously taught enhanced their ability to cope, concentration, sense of calm as well as facilitating other coping methods such as breathing and visualisation.	4 studies: [23, 26, 39, 42]	Low	*Impact on focus and capabilities*
3. Relaxation techniques facilitated a positive labour and birth with effectiveness as a pain relief: Relaxation techniques had several positive purposes such as creating a peaceful birthing environment. The techniques were an effective pain relief method, either by lessening the perceived levels of pain or by making the contractions more bearable. Additionally, the women reported feelings of relaxation and an empowered sense of control. Together, this facilitated positive feelings regarding the labour and birth.	7 studies: [23, 26, 38, 39, 42–44]	Moderate	*Impact on pain* *Impact on focus and capabilities*
4. Positive way to ‘work with the pain’: The use of relaxation techniques reframed the women’s approach to pain to a positive model of ‘working with the pain’. This was effective either through increased levels of confidence and ability to cope or as means of distraction away from the pain.	4 studies: [24, 26, 42, 44]	Low	*Impact on pain*
5. Enhanced wellbeing during the birth and postnatal period: The relaxation techniques provided women with positive feelings of safety, strength, joy and connection. The women reported ongoing benefits throughout the postnatal period such as soothing the baby, coping with parenting and facilitating breastfeeding.	5 studies: [23, 26, 38, 43, 44]	Moderate	*Impact on wellbeing and health*
6. Enhanced participation of birth companions and caregivers: Women reported that taught massage techniques provided their birth companions with the tools to participate in labour preparation and during the birth and enhanced their relationships with caregivers.	6 studies: [24, 38, 39, 42–44]	Low	*Influence and experience of support*
7. Valued variety of techniques: Where women were taught a range of techniques during the antenatal period, they valued having a ‘toolkit’ they could use during the birth. In this way, they could adapt the techniques to meet their changing needs throughout labour and birth.	3 studies: [24, 26, 42]	Very low	*Impact on focus and capabilities*
8. Not always effective: For a minority of women the taught techniques were not always as effective as they anticipated in alleviating pain.	2 studies: [23, 44]	Very low	*Impact on pain*

### Themes arising from stage two analysis

Five themes emerged from stage two analysis. Theme one (*‘desires for pain relief’*) illuminates different reasons for using pharmacological or non-pharmacological pain relief. Theme two (*‘impact on pain’)* describes varying levels of effectiveness of the methods used. Theme three (‘*influence and experience of support’*) highlights women’s positive (pharmacological/non-pharmacological) or negative (pharmacological) experiences of support from professionals and/or birth companions. Theme four (‘*influence on focus and capabilities’*) illustrates that while all pain relief methods could help women feel in control, some found non-pharmacological techniques less effective than anticipated, and others disliked complications associated with medication use. The final theme (‘*impact on wellbeing and health’*) reports that whilst some women were satisfied with their pain relief method, medication was associated with feelings of guilt and failure, whereas women taught relaxation techniques often continued to use these methods with beneficial outcomes. These themes are discussed in more detail below.

### Desire for pain relief

Some women made the decision to have an epidural analgesia (EA) in labour while they were pregnant [18, 27–32]. This decision was based on a woman’s previous positive experience of EA [33], or a negative experience of a medication-free labour:

*‘I’m not into pain, I wanted an epidural. I spent most of the time screaming, “I want an epidural; I’m not doing this, I’m going home!” I gave birth completely natural with no medication whatsoever, and I was hysterical. I did not want that. I don’t like pain, and it hurts very bad, and I don’t understand why any woman would want to birth naturally’* (p.30) [28].

Other pre-birth desires for an EA related to the messages received via the media, health professionals (e.g. at antenatal classes) or social network members. Stories of insufferable pain or complications instilled fear, where women felt ‘*warned to have* [an] *epidural’* (p.7) [18]. Whereas positive accounts provided encouragement [28–30]:

*‘I heard from a woman who had a childbirth earlier than me and had an epidural. A (Japanese) woman who experienced epidural anesthesia told me that it was comfortable and the experience was good. So I was wondering if I should try it for my second baby’* (p.313) [29].

The widespread availability of EA (and associated perceptions of safety) also served to normalise EA as an expected and safe intervention [28, 29, 34, 35]:

*‘I think like 75 or 85 percent of women have epidurals now. Ok? It’s pretty common to have epidurals, and they talked a lot about it... That definitely opened my eyes to considering an epidural’* (p.191–192) [35].

Common reasons for a pre-birth desire for EA were fear of pain, a need to remain in control and wanting a pain-free labour [18, 27, 28, 31, 34]:

*‘I don’t like pain as any human, and if I can avoid it without harm to my baby I did just that’* (p.328) [27].

For other women their decision to have an EA [27, 29, 32–34, 36] or opioids [37] was made during the intrapartum period. These women opted to receive medication at a critical point in their labour when they felt out of control, depleted of energy, and the level of pain was intolerable and unmanageable:

*‘…I was just like almost on the floor, like it* [the pain] *was really bad…you don’t want to overreact, but it is so much pain that you do not know what to do’* (p.473) [33].

The relaxation and massage studies did not generally describe why women sought these methods of pain relief. This was often because participating women used/were taught these methods as part of their involvement in a research study. However, a woman from Klimi et al’s [26] music therapy study offered a different perspective than those who planned to use an EA. She described how music coalesced with her desire for a tranquil, intervention-free birth:

*‘I wanted a familiar environment with my own people, music, tranquillity….Let my baby come to life calmly, without medication and, of course, with a normal childbirth’* (p. 300) [26].

Overall, there were similarities and differences in how the different methods of pain relief influenced women in the pre-birth period. For some women, knowing an EA was available helped to alleviate fears and provided a sense of reassurance [18, 33, 34]:

*‘A good thing* [in New Zealand] *is that an epidural is right there as an option. In Japan, Since the place I lived was in the country, there was nowhere and no hospital to do such a thing. I was thinking that I really did not want to give birth in Japan because I was sensitive to pain’* (#9 p.123) [34].

Similarly, women who received antenatal training in massage and/or relaxation methods referred to how knowledge of these pain-relief methods provided a sense of relief [23, 24]:

*‘I think for me it was the acupressure, knowing that there was something that could help without drugs or an epidural’* (Mia, p.128) [24].

Training in the use of non-pharmacological techniques enabled women to feel ‘*prepared’*, *‘calm’* and *‘empowered’* for childbirth [24, 38, 39]:

*‘So I found that this workshop* [MBCE] *gave me a lot more empowerment and a lot more information about alternate courses of action and different scenarios, so I’d be prepared* [during labour]*’* (mother 3, p.6) [39].

Some women used the relaxation techniques in the antenatal period to reduce childbirth fears: ‘*When I got really worried about the birth, I would just breathe to stop my mind from going all sorts of bad places’* (p.198) [38]. Whereas women who had made a decision for an EA worried about needle placement, ineffectiveness and negative implications [28, 29, 33, 35, 36]:

*‘I remember worrying because of what I heard about the use of a big needle, and the risks and complications’* (p27) [36].

### Impact on pain

Overall, there were conflicting accounts on the effectiveness of the different pain relief methods in alleviating pain. A number of studies reported that EA [18, 27, 30, 36, 40] and opioids [41] had a positive influence on pain. One study [41] involved interviews with women who had been randomly allocated to three different forms of opioid-based pain relief; intramuscular pethidine, intranasal remifentanil and subcutaneous fentanyl. Some women from each of the different intervention arms expressed how the medication had had a positive impact on pain and/or shortened and reduced the intensity of the contractions. Patient-administered medication enabled women to feel in control over their labour pain [33, 41].

In contrast, others stated that the EA [27, 33, 35, 40] or opioids [18, 37, 41] had been ineffective. Negative accounts of pethidine were reported across all the studies (*n* = 3) – where women described it as *‘useless’* and how ‘[it] *did not work’* (p.6) [18]:

*‘When I get the pain then I think to myself I got the injection* [pethidine]*, why am I still getting pain’* (p.87) [37].

Some women in the Fleet et al. [41] study did not find the intranasal fentanyl to be helpful in managing their labour pain, but when women compared intranasal fentanyl with their experience of intramuscular pethidine, they reported that intranasal fentanyl was more effective:

*‘I felt really out of it. At the time I wasn’t sure if the intranasal fentanyl was still helping but after using the Pethidine I was more aware that it had been, without causing the high or sedation’* (p.18) [41].

Some of the difficulties associated with pharmacological methods related to women experiencing pain at needle citation [40], breakthrough pain (i.e. where women continue to experience pain or pressure following EA citing) [33], the half-life of the medication [35, 40, 41], and epidurals needing to be placed more than once [35, 40]. Other issues related to delays in receiving the medication, or the medication being provided too late to be effective [33, 40]:

*‘I was hoping to get* [the epidural] *right away, but when they told me 30 minutes, I give up…so I started to scream’* (p. 473 multiparous) [33].

Variations in effectiveness of non-pharmacological methods in reducing labour pain was also noted. Some women who used relaxation and/or massage [23, 25, 26, 38, 39, 42–44] techniques reported how these methods helped to make the pain more bearable. Women who received music therapy highlighted how *‘it hurt less’* (p.445) when the music was playing and how their pain increased during the planned 2-h non-music intervals [43]. Massage techniques also helped to reduce the contractions and make them easier to manage and provided women with a *‘lifeline’* to cope:

*‘Where the pains overwhelm me and I feel like falling into a void and getting lost, the music was exactly like this: A lifeline that somewhere I was saying, say, something I recognized, gave me strength and I continued ...’* (p.302) [26].

However, a few women in the non-pharmacological studies reported how these methods had been less effective than anticipated [23, 44, 45]: ‘[but toning - extended vocal *sounds* on a single vowel] *did not feel as effective as I’d hoped during labour’* (p.221) [44]. Difficulties related to the intensity of the pain, and subsequent loss of control; ‘*No, no control… intense pain, emotionally exhausted; I was not in control at all’* (p.4) [23]. Other criticisms related to the volume of the music (i.e. not loud enough), or music stopping at crucial points [26, 42, 43]. Women in one study [26] also complained of distractions when hearing sounds other than the music, e.g. traffic.

### Influence and experience of support

Some of the women who received EA valued the fact that health providers had respected and supported their pain relief choice [28–30, 33, 35, 40]; a position considered important to prevent against maternal guilt:

*‘Actually the nurse and the doctor came by a few times and because they say I was really suffering, they said “You know if you want it (the epidural) it’s okay”. I thought that that was wonderful. It makes you feel better, ‘cause I think there is often a tendency to make you feel guilty…You are the one, you know, going through the labour’.* (p. 474 multiparous) [33].

However, others reported on how healthcare staff had *‘hounded’* or pressured women into having an EA [27, 28, 34, 35, 40], such as through instilling unnecessary fears:

*‘But my midwives recommended that I should use epidural by telling me that I had been doing my best and that the baby’s health is the most important although the baby had not shown any problems’* (p.123) [34].

In Morris & Schulman’s [35] study they report how women from an ethnic minority background and low education were more likely to experience ‘pressure’ from clinicians to receive an EA. This was due to being more likely to be induced, and through offering ‘false’ choice: ‘*The nurse asking me – did I want to go have a C-section or get the epidural shot?’* (p.193).

Women within the pharmacological and non-pharmacological studies reported how standard, traditional pre-natal classes focused on, or promoted medication [18, 28, 30, 35]. There were also occasions of a lack of consent in the pharmacological studies. A woman who received pethidine in Jantjes et al.’s study [37] stated; *‘the doctor just walked in and said they are going to give me an injection’* (p.87–88). While some women felt well informed about procedures and risks for EA use [29], for others, this information was lacking [28, 32, 34, 36, 40]:

*‘My blood pressure dropped. The baby’s heart rate dropped to [the] seventies. I’ve heard a lot about epidurals from television and friends, but I didn’t know that could happen’* (p.29) [36].

Accounts of women feeling pressured or ill-informed were not evident within the non-pharmacological studies. There were, however, variations in how the pain relief methods influenced women-birth supporters’ relationships. Women who used relaxation or massage techniques frequently recounted how these methods had encouraged and enabled connections with their healthcare providers and/or birth companions [24, 25, 38, 39, 42–44]:

*‘I felt very connected to my partner, the class taught us how to work as a team and be fully present in the moment and that connection got me through delivery and the post-partum period’* (p.198) [38].

Non-pharmacological methods appeared to facilitate teamwork, a *‘bond’*, with their birth companions, which in turn induced a sense of security, calmness and being cared for [23–25, 38, 42–45]; *‘It was felt that I was not alone and I felt more relaxed’* (p.12) [25].

Some women using pharmacological pain relief reported positive interactions with caregivers and birth companions following administration of analgesia [32, 36]. More commonly, were accounts of negative women-provider relationships. Some women blamed their choice of pain relief (i.e. EA) on insufficient nursing support [33]. Other women expressed negativity due to: lack of caregiver support for their choice of pain relief [32, 33]; being reliant on staff to administer ongoing pain relief [40, 41]; or how it had alienated them from their care providers [36, 37, 40]. Some expressed that interactions with care providers became more distant after they chose to receive pharmacological pain relief, particularly when there was no continuity of care; *‘The second midwife, she came in when I was totally doped – there was no contact’* (p.101) [32]. These experiences thereby indicate how the use of medication replaced the availability of personal care:

*‘I didn’t want to go on anymore as I was alone and there was nobody to support me…..I needed somebody at that time just to hold onto’* (p.87) [37].

### Influence on focus and capabilities

Overall there were similar accounts in women’s experiences of the different pain relief methods in how they had enabled them to relax, feel calm, and in control [18, 23–26, 28, 30–33, 36, 38, 39, 41–44]:

[in context of receiving an EA]*‘You’re kind of euphoric for a second. All pains are gone. You aren’t tensed up anymore. You are relaxed and feel so much better. You can still feel some pressure of contractions, but you don’t have constant pain going through your entire body’* (p.27) [36].

*‘It* [mindfulness] *provided me with....a sense of calm and a sense of being in control, even though everything around me was out of control’* (p.6, mother 7) [39].

A means to eradicate or manage their pain enabled women to rest and to restore and re-focus their energy [23–26, 32, 33, 41]; ‘*Maybe it* [epidural analgesia] *was a regain of control – I got new energy’* (p.100) [32]:

*‘While I was pushing, once the tone got high and frantic. I could tell that I was tensing and not relaxing. Bringing the tone down low and slowing it down helped me feel relaxed and open again’* (p.220) [44].

A number of the women, irrespective of the pain approach used, considered that their pain relief method had been crucial for them to achieve a vaginal birth [18, 27, 30, 36, 43–45]:

‘*I would never have done it* [given birth] *without the epidural’* (p.6) [18].

*‘I could not have done what I did without music’* (p.445) [43].

A key difference between the pharmacological and non-pharmacological methods concerned how they directed women’s attention, focus and capabilities. From a negative perspective, pharmacological methods were reported to have *‘slowed the labour down’* [41]; negatively impacted on women’s ability to push [27, 40, 41] and for women to feel disconnected from the baby and childbirth [32, 41]. For some women, a lack of mobility (following EA citing) induced discomfort, anxiety, fear [33, 36], whereas pethidine led to cognitive distortions [37, 41]:

*‘The Pethidine knocked me out, didn’t help with the pain. Made me sleep between contractions but wasn’t a good experience’* (p.18) [41].

Pharmacological pain relief caused side effects such as nausea, numbness, itching, coldness and a decrease in blood pressure [33, 37, 40, 41].

However, when EA was effective, some women described how they were able to focus on the external environment. Once women’s physiological and emotional responses had been stabilised, they could observe what was happening, focus on the baby, and regain participation [28, 31–33, 36]:

*My body was only concentrated in pain. It was almost like I was not in the present. Once the pain was gone, I was able to concentrate on [the experience], concentrate on my husband, my sister, my nurse, the doctor. I could hear what [they] said, and [understood] what I needed to do. I do not think all those things would have been possible without the epidural.*’ (p.28) [36].

While massage techniques could not always be effectively applied, e.g. due to maternal position [23], women who used non-pharmacological methods recounted how the techniques provided a distraction that enabled women to *‘face up to’* (p.40) [45] and *‘release into the pain’* (p.221) [44] and manage their contractions. Their focus turned inwards as they flexibly and actively worked with their bodies through using taught or adapted techniques [23–25, 42, 44, 45]:

*‘The breathing exercises, the massages, the baths, and then, I did everything, and the positions I adopted… Because if I just stayed lying down, then the pain felt even worse; then when I sat up in that butterfly position or with my two feet together, I could put more effort into it when it contracted, and with my breathing, I could relax, and when I was able to relax, the pain was less.* (24 years old p.4) [23].

### Impact on wellbeing and health

Many of the women who used pharmacological or non-pharmacological methods expressed positive feelings towards their chosen method. Some women who used pharmacological pain relief reported how their fears had been unfounded [28, 36] and expressed gratitude as EA had enabled them to enjoy their birth experience [27, 30, 31, 33, 36, 40]. For women who received non-pharmacological pain relief, this was expressed as feelings of control, joy and empowerment [23–26, 38, 39, 42, 44, 45]. However, some women irrespective of which method of pain relief used reported more ambivalent responses, albeit for different reasons. For instance, a few women who used medication reported how their initial disappointment eventually dissipated into acceptance [32, 34, 36, 40]:

*‘I originally wanted to give birth without an epidural, but changed my mind about 14 hours after labor began. For a while I felt a little guilt about “giving in” but came to realize that each labor is different and a “woman’s got to do what a woman’s got to do”’* (p.6) [40].

Whereas one woman in Kimber’s [45] study held a more equivocal opinion in that while the method had helped her to manage their pain, she had been unable to continue its use due to labour complications:

*‘Very useful as a means of pain relief. Used for the first ten hours with breathing techniques as the sole means of relief. It proved very good and I feel it would have been possible to rely on massage, had I not failed to progress for the entire labour’.* (nullipara p.40) [45].

However, unlike the experiences reported in the non-pharmacological studies, a number of women who used medication reported negative self-reprisals, such as feeling guilty and a failure [32–34, 37, 41], which for some, as reflected by a Japanese mother, was related to pharmacological methods not being her cultural norm:

*‘….I cried because of guilt to the midwife and my husband. I felt like “I am sorry I did not try hard enough” and “I am sorry I failed”’* (p.123) [34].

Some of the women who had used an EA as a method of pain relief referred to ongoing ‘*back problems*’ [27] and held fears over potential future complications [33]. One woman who had used pethidine also reported how it had affected her postnatal memory recall:

*‘Don’t even remember the early period after birth, looked at photos and didn’t remember it happening*’ (P.137, p.18) [41].

A stark point of difference between the pain relief methods related to how mothers continued to use the relaxation techniques in the postnatal period, with positive impacts for mothers and infants [26, 38, 39, 42, 43]. Women referred to how they used the techniques to help deal with the demands of new motherhood:

*‘I have also used mindfulness to notice when emotions crop up such as feeling overwhelmed, sad, or resentful of my husband as he sleeps and I get up in the wee hours to nurse. Instead of reacting to these emotions I’m able to just note them in a non-judgmental way. From there I can either think through what made me feel that way or bring them up and talk with my husband about them’* (p.198) [38].

Women within the music studies also referred to how music was used as an effective means to settle and soothe their baby [26, 42, 43], and to facilitate breastfeeding:

*‘yesterday, she wouldn’t latch on properly, and she was a little, um, finicky, and we put the music on and right away she latched on, she fed, she had a good feeding and then she went to sleep right away. It was great! It was amazing the difference it made to her…’* (p.275) [42].

## Discussion

Overall, the findings revealed mixed experiences of the pharmacological (epidurals and opioids) and non-pharmacological (massage, relaxation) methods of pain relief included in the review. In terms of pharmacological pain relief, planned use of these methods in labour was initiated by negative previous experiences of labour pain by some, and by positive previous experiences of using these methods by others. Some did not plan to use these methods when they were pregnant, but did so once in labour. This was sometimes related to the unexpected intensity of labour pains, but it was notable that women using these methods were more likely to recount negative experiences of health provider support. Given the association between labour support and decreased labour pain that is evident in clinical trials, poor caregiver support, in contrast, could be a factor in unanticipated need for pharmacological pain relief. Women in this group were also more likely to report feelings of guilt and failure due to their unexpected need to use medication for labour pain relief. Many of the women who used non-pharmacological methods more likely to express beliefs aligned with a natural physiological approach, and their accounts suggested that they felt prepared for childbirth. In contrast to the women using pharmacological methods, those who used non-pharmacological techniques referred to how these methods encouraged and facilitated positive support from health providers and birth companions. Pharmacological and non-pharmacological pain relief methods had the potential to help women feel in control. However, women reported negative effects of both. Some women who used using relaxation/massage techniques reported them to be less effective than anticipated, but others who had been taught relaxation methods continued their use in the postnatal period, with reported positive effects for themselves, their babies and/or their families. Overall, the findings offer some support to the recent effectiveness systematic review of reviews of methods for labour pain relief [5] in which, while epidurals were the most effective approach to alleviate pain, they were associated with more adverse effects, and lower rates of satisfaction. In the same review, while relaxation/massage techniques were not necessarily effective for pain relief, they were more likely to be associated with other positive outcomes.

The strengths of this study are that a comprehensive and rigorous search strategy was undertaken. We also took care to capture and reflect variations across the studies, such as through including non-English papers. While a qualitative evidence synthesis is an interpretative process, the risk of over or under interpretation of the data was minimized through author reflexivity to ensure that personal beliefs and values did not obscure important data within the included studies, and through rigor in study selection and analysis. There were however limitations. First, the review focused on specific pain relief techniques, and others e.g. acupuncture, sterile water injections were not included. There is no guarantee that we captured all published studies in our search strategy. We found few studies that related to women’s experiences of opioids [18, 37, 41] or massage techniques [23–25, 45], and none of the studies were undertaken in low-middle income countries. Furthermore, as only four of the included studies were published in the last five years, this suggests that more contemporary insights should be sought. Some of the relaxation/massage studies also combined different techniques, which made it challenging to differentiate between the approaches. Few studies focused upon women from marginalised populations i.e. low education, teenage parents, ethnic minorities, thereby limiting the generalisability of the findings.

Our findings support those of others that coping with the pain of childbirth is complex and multifaceted [46]. In our review, while all the included studies were from upper-middle/high income countries where the use of pharmacological methods of pain relief is common practice, we identified a wide range of preferences for pain relief. In particular, there was divergence between those who did and who did not plan to use medication in labour. Some women appeared to hold an uncritical acceptance of epidural analgesia, due its widespread use and associated perceptions of safety [27–30, 32–35]. Use of pharmacological pain relief was associated with increased control by some [27, 28, 30, 31, 34, 41]. Acceptance of intrapartum interventions that reduce and control the uncertainties and discomfort of childbirth has been described in other studies [31, 47–49]. However, as Lally noted [31], in this review, many of those who preferred not to use pharmacological pain relief ended up with it anyway, and this group of women were particularly likely to express feelings of guilt and failure. Women’s attitudes towards technical solutions for pain relief are inevitably influenced by cultural norms that value technological progress, and that present particular solutions as mainstream and freely available.

Where the methods used fitted with the a priori frame of reference of the woman, or where they accepted that the uncertainties of labour were the basis for using methods they might not have chosen, women seemed to find whatever method(s) they used effective in enabling them to relax and regain a sense of control over the birth. However, there were notable differences between the pain relief approaches related to how they directed women’s attention and focus. Pharmacological methods, particularly when effective, enabled women to focus on the external environment to converse with others and observe their labour progress objectively [28, 31–33, 36]. In contrast, non-pharmacological methods were associated with an internal focus, in which the women seemed to be more actively engaged with and responsive to their body as it experienced the dynamic physiological responses of labour over time [23, 24, 42, 44, 45]. As Leap et al. have noted, this is the difference between ‘pain relief’, and ‘working with pain’ [50].

A key finding from our review was how the need for/availability of social support differed for women using medication and those who used other methods. Specifically, our findings extend those by Jones et al. [5] in that the efficacy and satisfaction of the chosen methods related to the quality of the mother-midwife relationship. In our review, women reported mixed feelings regarding their caregivers in relation to their decision to have an epidural and/or opioids. Some women who received medication felt supported in their choice [28–30, 33, 35, 40]. However, more commonly there were complaints of women feeling pressured to receive medication, lack of consent/information on risks, tension due to opposing women-provider views of medication use, or women being left unattended after medication administration [27, 28, 32, 34–37, 40]. Relaxation and massage techniques, on the other hand, facilitated meaningful and connected women-provider relationships [24, 38, 39, 42–44]. This approach aligns with a midwifery philosophy of continuous, woman-centred care [51] to facilitate the biopsychosocial physiology of childbirth [12]; with maternity professionals who use complementary therapy approaches referring to how they promoted confidence and pride in their profession [24, 52, 53]. As relational care, and in particular, continuous care during labour is associated with positive outcomes (increased vaginal births, fewer interventions, fewer pharmacological pain relief) and increased levels of women’s satisfaction [54, 55], it should be provided irrespective of the type of pain relief method used. Efforts to promote labour support may help to improve women’s coping skills [46, 56, 57], and helping women to avoid medication if this is their preference could also result in clinical benefits for the mother and/or baby [26, 39, 42, 43]. While further research is needed, the insights suggest that non-pharmacological methods, even in combination with pharmacological methods, could be beneficial for women and providers.

Survey data from low income settings indicate that women’s access to and knowledge of pharmacological pain relief is low [58–60]. Where women do know about these options, similar variation in values and beliefs are evident as in the current review. Some women believe in the need for effective pain relief [58, 61], and others placed intrinsic value on the experience of ‘natural’ childbirth [59, 60]. Our findings from high and middle income settings, and those for low income settings from survey data, support the observation that choices for pain relief are influenced by cultural [62] and personal factors [63] as to how childbirth is perceived. Women may be more likely to opt for medication if they view childbirth as a medical condition with risks, whereas those who view it as a normal, natural event may be more likely to use natural, or non-pharmacological approaches [63]. A more recent innovation designed to address the shortcomings of numerical pain rating scales, that recognises the complexity and multifaceted nature of pain management, and to offer support aligned with women’s needs during childbirth is the Coping with Labor Algorithm [64, 65]. The tool involves asking women how they feel they are coping, with detailed cues (psychological, behavioural, physiological) used by maternity professionals to elicit whether the women is coping well (or not coping) with labour. Depending on these assessments, continued support (if coping well) or specific interventions (if not coping well) are offered that include different pain relief methods, changes to the physical environment and additional emotional support (Roberts et al., 2010). Maternity care professionals’ evaluations of the tool have been very positive e.g. in enhancing respectful, woman-centred care [64, 66], although further work to test its efficacy on women’s experiences is needed.

## Conclusion

Women have mixed experiences of different pharmacological and non-pharmacological pain relief methods. Women varied in their opinion as to whether the different pain relief methods were effective in reducing their labour pain. The different pain relief approaches could enable women to relax and feel in control. However, women who used medication were more likely to experience negative side effects, negative encounters with healthcare providers, and a sense of guilt and/or failure. While non-pharmacological methods did not necessarily reduce labour pain or facilitate a vaginal birth, they could enable women to actively work with their physiological responses and facilitate a ‘team’ approach with their birth supporters. Continued use of relaxation methods in the post-natal period by some women also provided benefits for them, their babies, and their families, suggesting that learning these techniques provided the basis for self-help in the longer term. The findings highlight the need for women to receive complete information on the risks as well as the benefits of the range of methods that will be available to them in labour. They also highlight a need to promote and provide as many approaches as possible, so that women have access to methods that meet with their prior values and beliefs, as well as to those that they may need if their experiences differ from their expectations. The value of social support in labour should be recognised by funders and providers, and prioritised in service provision and staffing, as this was seen as valuable by women from both groups.

## References

[1] Whitburn L, Jones L, Davey M, Small R (2017). The meaning of labour pain: how the social environment and other contextual factors shape women’s experiences. BMC Pregnancy and Childbirth.

[2] Karlsdottir S, Halldorsdottir S, Lundgren I (2014). The third paradigm in labour pain preparation and management: the childbearing woman's paradigm. Scand J Caring Sci.

[3] Jones L, Whitburn L, Davey M, Small R (2015). Assessment of pain associated with childbirth: Women’s perspectives, preferences and solutions. Midwifery.

[4] Lundgren I, Dahlberg K (1998). Women's experience of pain during childbirth. Midwifery.

[5] Jones L, Othman M, Dowswell T, Alfirevic Z, Gates S, Newburn M, Jordan S, Lavender T, Neilson J. Pain management for women in labour: an overview of systematic reviews. Cochrane Database Syst Rev. (2012, 3). 10.1002/14651858.CD009234.pub2 PMC7132546 22419342

[6] Anim‐Somuah M, Smyth RMD, Howell CJ. Epidural versus non‐epidural or no analgesia in labour. Cochrane Database Syst Rev. 2005;4. Art No.: CD000331. 10.1002/14651858.CD000331.pub2. 10.1002/14651858.CD000331.pub2 16235275

[7] Sprawson E (2017). Pain in labour and the intrapartum use of intramuscular opioids- how effective are they?. Br J Midwifery.

[8] Burchell T, Coster S, Norman I (2016). The effect of intrapartum pethidine on breastfeeding: a scoping review. Evidence Based Midwifery.

[9] Littleford J (2004). Effects on the fetus and newborn of maternal analgesia and anesthesia: a review. Can J Anesth.

[10] Chaillet N, Belaid L, Crochetiere C, Roy L, Gagne G, Moutquin J, Rossignol M, Duga M, Wassef M, Bonapace J (2014). Nonpharmacologic approaches for pain management during labor compared with usual care: a meta-analysis. Birth: Issues in Perinatal Care.

[11] Smith CA, Levett KM, Collins CT, Armour M, Dahlen HG, Suganuma M. Relaxation techniques for pain management in labour. Cochrane Database Syst Rev. 2018;3. Art. No.:CD009514. 10.1002/14651858.CD009514.pub2. 10.1002/14651858.CD009514.pub2 PMC6494625 29589650

[12] Downe S, Finlayson K, Tunçalp Ӧ, Metin Gülmezoglu A (2016). What matters to women: a systematic scoping review to identify the processes and outcomes of antenatal care provision that are important to healthy pregnant women. BJOG Int J Obstet Gynaecol.

[13] Downe S, Walsh D, Simpson L, Steen M: Template for metasynthesis. 2009, Contact: sdowne@uclan.ac.uk.

[14] Lewin S, Glenton C, Munthe-Kaas H, Carlsen B, Colvin C, Gülmezoglu M (2015). Using qualitative evidence in decision making for health and social interventions: an approach to assess confidence in findings from qualitative evidence syntheses (GRADE-CERQual). PLoS Med.

[15] Thomas J, Harden A (2008). Methods for the thematic synthesis of qualitative research in systematic reviews. BMC Health Serv Res.

[16] Noblit G, Hare R (1988). Meta-ethnography: synthesising qualitative studies.

[17] Walsh D, Downe S (2006). Appraising the quality of qualitative research. Midwifery.

[18] Larkin P, Begley C, Devane D. Women's preferences for childbirth experiences in the republic of Ireland; a mixed methods study. BMC Pregnancy and Childbirth. 2017;17(1):19. 10.1186/s12884-016-1196-1. 10.1186/s12884-016-1196-1 PMC5223453 28068948

[19] Mizuo C, Shiono E (2013). Experiences leading up to childbirth by women who chose to have a painless delivery during pregnancy. Journal of Japan Academy of Midwifery.

[20] Nikkola E, Läärä A, Hinkka S, Ekblad U, Kero P, Salonen M (2006). Patient-controlled epidural analgesia in labor does not always improve maternal satisfaction. Acta Obstet Gynecol Scand.

[21] Fyneface-Ogan S, Mato C, Anya S (2009). Epidural anesthesia: views and outcomes of women in labor in a Nigerian hospital. Annals of African Medicine.

[22] Hasanzahraei R, Mehran N, Fathizadeh N, Abedi H (2007). Epidural painless delivery: a phenomenology research. Iran J Nurs Midwifery Res.

[23] Miquelutti M, Cecatti J, Makuch M. Antenatal education and the birthing experience of Brazilian women: a qualitative study. BMC Pregnancy and Childbirth. 2013;13:171. 10.1186/1471-2393-13-171. 10.1186/1471-2393-13-171 PMC3766656 24007540

[24] Levett K, Smith C, Bensoussan A (2016). Dahlen H: he complementary therapies for labour and birth study making sense of labour and birth- experiences of women, partners and midwives of a complementary medicine antenatal education course. Midwifery.

[25] Andrén K, Störholt L, Lundgren I (2005). Women's experiences of tactile massage during the latent phase of labour. Nordic Journal of Nursing Research.

[26] Klimi A, Economidou E, Froudaki M, Mantoudi A (2011). Music as a conditioning aid in the childbirth experience: a qualitative study. Hellenic Journal of Nursing.

[27] Heinze S, Sleigh M (2003). Epidural or no epidural anaesthesia: relationships between beliefs about childbirth and pain control choices. Journal or Reproductive and Infant Psychology.

[28] Dillaway H, Brubaker S (2006). Intersectionality and childbirth: how women from different social locations discuss epidural use. Race, Gender and Class.

[29] Yoshioka T, Yeo S, Fetters M (2012). Experiences with epidural anaesthesia of Japanese women who had childbirth in the United States. Journal of Anaesthesia.

[30] Goldberg H, Shorten A (2014). Patient and provider perceptions of decision making about use of epidural analgesia during childbirth: a thematic analysis. J Perinat Educ.

[31] Lally J, Thomson R, MacPhail S, Exley C. Pain relief in labour: a qualitative study to determine how to support women to make decisions about pain relief in labour. BMC Pregnancy and Childbirth. 2014;14:6. 10.1186/1471-2393-14-6. 10.1186/1471-2393-14-6 PMC3893516 24397421

[32] Jepson I, Keller K (2014). The experience of giving birth with epidural analgesia. Women and Birth.

[33] Angle P, Landy C, Charles C, Yee J, Watson J, Kung R, Kronberg J, Halpern S, Lam D, Lie L, Streiner D (2010). Phase 1 development of an index to measure the quality of neuraxial labour analgesia: exploring the perspectives of childbearing women. Canadian Journal Anaesthesia.

[34] Doering K, Patterson J, Griffiths C (2014). Japanese women's experiences of pharmacological pain relief in New Zealand. Women and Birth.

[35] Morris T, Schulman M (2014). Race inequality in epidural use and regional anaesthesia failure in labor and birth: an examination of women's experience. Sexual and Reproductive Healthcare.

[36] Hidaka R, Callister L (2012). Giving birth with epidural analgesia: the experience of first-time mothers. J Perinat Educ.

[37] Jantjes L, Strumpher J, Kotze W (2007). The experience of childbirth in first-time mothers who received narcotic analgesics during the first stage of labour. Curationis.

[38] Duncan L, Bardacke N, Duncan LG, Bardacke N (2010). Mindfulness-based childbirth and parenting education: promoting family mindfulness during the perinatal period. J Child Fam Stud.

[39] Fisher C, Hauck Y, Bayes S, Byrne J: Participant experiences of mindfulness-based childbirth education: a qualitative study. BMC Pregnancy and Childbirth 2012, 12(1):126–126. 10.1186/1471-2393-12-126 PMC3534482 23145970

[40] Attanasio L, Kozhimannil K, Jou J, McPherson M, Camann W (2015). Women's experiences with neuraxial labor analgesia in the listening to mothers II survey: a content analysis of open-ended responses. Anaesthesia and Analgesia.

[41] Fleet J, Jones M, Belin I (2017). Taking the alternative route: Women's experience of intranasal fentanyl, subcutaneous fentanyl or intramuscular pethidine for labour analgesia. Midwifery.

[42] Browning C (2000). Using music during childbirth. Birth: Issues in Perinatal Care.

[43] Tabarro C, de Campos L, Galli N, Novo N, Pereira V (2010). Effect of the music in labor and newborn. Rev Esc Enferm USP.

[44] Pierce B (2001). Toning in pregnancy and labor. The Journal of Prenatal and Perinatal Psychology and Health.

[45] Kimber L (1998). Reflective practice. How did it feel? An informal survey of massage techniques in labour. The Practising Midwife.

[46] Van der Gucht N, Lewis K (2015). Women’s experiences of coping with pain during childbirth: a critical review of qualitative research. Midwifery.

[47] McAra-Couper J, Jones M, Smythe L (2011). Caesarean-section, my body, my choice: the construction of ‘informed choice’ in relation to intervention in childbirth. Fem Psychol.

[48] Cherniak D, Fisher J (2008). Explaining obstetric interventionism: technical skills, common conceptualisations, or collective countertransference?. Women's Stud Int Forum.

[49] Kornelsen J, Hutton E, Munro S (2010). Influences on decision making among primiparous women choosing elective caesarean section in the absence of medical indications: findings from a qualitative investigation. J Obstet Gynaecol Can.

[50] Leap N, Anderson T, Downe S (2008). The role of pain in normal birth and the empowerment of women. Normal childbirth: evidence and debate.

[51] Renfrew M, McFadden A, Bastos M, Campbell J, Channon A, Cheung N, Silva D, Downe S, Kennedy H, Malata A, McCormick F, Wick L, Declercq E (2014). Midwifery and quality care: findings from a new evidence-informed framework for maternal and newborn care. Lancet.

[52] Mollart L, Adams J, Foureur M: 2016. Pregnant women and health professional’s perceptions of complementary alternative medicine, and participation in a randomised controlled trial of acupressure for labour onset. 24, pp.167–173. Complementary therapies in clinical practice 2016, 24:167–173. 10.1016/j.ctcp.2016.06.007 27502818

[53] Williams J, Mitchell M (2007). Midwifery managers’ views about the use of complementary therapies in the maternity services. Complement Ther Clin Pract.

[54] Hodnett E (2002). Pain and women’s satisfaction with the experience of childbirth: a systematic review. American Journal of Obstetrics and Gynaecology.

[55] Sandall J, Soltani H, Gates S, Shennan A, Devane D. Midwife-led continuity models versus other models of care for childbearing women. Cochrane Database Syst Rev. 2016;4. Art. No.: CD004667. 10.1002/14651858.CD004667.pub5. 10.1002/14651858.CD004667.pub5 PMC8663203 27121907

[56] Nikula P, Laukkala H, Pölkki T (2015). Mothers' perceptions of labor support. MCN: The American Journal of Maternal Child Nursing.

[57] Simon R, Johnson K, Liddell J (2016). Amount, source, and quality of support as predictors of women's birth evaluations. Birth: Issues in Perinatal Care.

[58] Nabukenya M, Kintu A, Wabule A, Muyingo M. Kwizera: knowledge, attitudes and use of labour analgesia among women at a low-income country antenatal clinic. BMC Anesthesiol. 2015;15:98. 10.1186/s12871-015-0078-9 10.1186/s12871-015-0078-9 PMC4492001 26148501

[59] Naithani U, Bharwal P, Chauhan S, Kumar D, Gupta S (2011). Kirti: knowledge, attitude and acceptance of antenatal women toward labor analgesia and caesarean section in a medical college hospital in India. J Obstetric Anaesth Crit Care.

[60] Olayemi O, Aimakhu C, Udoh E (2009). Attitudes of patients to obstetric analgesia at the university college hospital, Ibadan, Nigeria. J Obstet Gynaecol.

[61] Audu B, Yahaya U, Bukar M, El-Nafaty A, Abdullahi H, Kyari O (2009). Desire for pain relief in labour in northeastern Nigeria. Journal of Public Health and Epidemiology.

[62] Callister L, Khalaf I, Semenic S, Kartchner R, Vehvilainen-Julkunen K (2003). The pain of childbirth: perceptions of culturally diverse women. Pain Management Nursing.

[63] Fenwick J, Hauck Y, Downie J, Butt J (2005). The childbirth expectations of a self-selected cohort of Western Australian women. Midwifery.

[64] Roberts L, Gulliver B, Fisher J, Cloves K (2010). The coping with labor algorithm: an alternate pain assessment tool for the laboring woman. Journal of Midwifery and Women's Health.

[65] Howard E (2017). An innovation in the assessment of labor pain. J Perinat Neonatal Nurs.

[66] Fairchild E, Roberts L, Zelman K, Michelli S, Hastings-Tolsman M (2017). Implementation of Robert's coping with labor algorithm in a large tertiary care facility. Midwifery.

